# The Examination of the Effects of Writing Strategy-Based Procedural Facilitative Environments on Students' English Foreign Language Writing Anxiety Levels

**DOI:** 10.3389/fpsyg.2016.02074

**Published:** 2017-01-10

**Authors:** Ioanna K. Tsiriotakis, Eleni Vassilaki, Ioannis Spantidakis, Nektarios A. M. Stavrou

**Affiliations:** ^1^Department of Primary Education, School of Education, University of CreteRethymnon, Greece; ^2^Faculty of Physical Education and Sport Science, National and Kapodistrian University of AthensAthens, Greece

**Keywords:** EFL writing anxiety, metacognition, strategy-based procedural facilitation, cognitive apprenticeship, procedural facilitative writing environments

## Abstract

Empirical studies have shown that anxiety and negative emotion can hinder language acquisition. The present study implemented a writing instructional model so as to investigate its effects on the writing anxiety levels of English Foreign Language learners. The study was conducted with 177 participants, who were administered the Second Language Writing Anxiety Inventory (SLWAI; Cheng, 2004) that assesses somatic, cognitive and behavioral anxiety, both at baseline and following the implementation of a writing instructional model. The hypothesis stated that the participant's writing anxiety levels would lessen following the provision of a writing strategy-based procedural facilitative environment that fosters cognitive apprenticeship. The initial hypothesis was supported by the findings. Specifically, in the final measurement statistical significant differences appeared where participants in the experimental group showed notable lower mean values of the three factors of anxiety, a factor that largely can be attributed to the content of the intervention program applied to this specific group. The findings validate that Foreign Language writing anxiety negatively effects Foreign Language learning and performance. The findings also support the effectiveness of strategy-based procedural facilitative writing environments that foster cognitive apprenticeship, so as to enhance language skill development and reduce feelings of Foreign Language writing anxiety.

## Introduction

### Anxiety

Learning a foreign language (FL) is a difficult task. One of the difficulties associated with foreign language learning is anxiety. Anxiety is a feeling of uneasiness and apprehension, usually in regards to a situation entailing uncertain outcomes (Spielberger and Gorsuch, 1983). During the last several decades, anxiety, among other affective variables, has stimulated attention in the field of second language acquisition and learning. Anxiety began to be closely observed in terms of how it facilitates or hinders language acquisition; a fundamental reason being that a noteworthy percentage of language learners alleged to possess difficulties while learning a foreign language. Specifically, language learners asserted that their difficulties were associated to an anxiety reaction impeding their ability to thrive in a foreign language setting (Horwitz et al., 1986). Hence, foreign language anxiety has been described as a negative emotional reaction to language learning (Horwitz, 2001). The learning process is determined by various factors such as the individual learners' metacognitive, cognitive abilities, personality characteristics, learning styles, learning strategies, social contexts, and affective aspects. The affective aspects that, in turn, deal with attitude, motivation, and anxiety, are also determinant factors in the learning process, with foreign language anxiety being considered a leading affective variable in foreign language acquisition (MacIntyre and Gardner, 1994b).

Anxiety is of vital importance in the field of foreign language learning as empirical research has determined anxiety to be an affective variable that inhibits the learning and/or production of a foreign language (Horwitz et al., 2010). In particular, foreign language anxiety has been depicted as a situation-specific anxiety related to foreign language learning that negatively influences performance (Horwitz, 2001). Essentially, foreign language anxiety is a universal phenomenon that ultimately hinders ESL/EFL achievement and that has consistently been identified as a key variable affecting performance in particular, and foreign language learning in general (Aida, 1994; MacIntyre and Gardner, 1994a). Both educators and researchers share concerns in terms of the negative effects of anxiety on foreign language learning and performance as well as the subtle effects of anxiety on language learning (MacIntyre et al., 1997).

### Foreign language (FL) anxiety

Early studies on foreign language anxiety approached foreign language learners' anxiety as either trait anxiety, a consistent personality trait that is pertinent across several circumstances or state anxiety, an anxiety experienced at a given particular moment (MacIntyre and Gardner, 1991a, p. 87). However, the trait or state approach failed to depict the nature of foreign language anxiety. Recent literature maintains the theory that foreign language anxiety is a particular language learning anxiety and a principal causative variable that restrains language acquisition (Horwitz, 2001).

Horwitz et al. (1986, p. 125) portray anxiety as a subjective feeling of tension, apprehension, nervousness, and even worry stimulated by an arousal of the autonomic nervous system. According to the latter, foreign language anxiety is explicitly connected with foreign language learning contexts and is regarded as a “distinct complex of self-perception, beliefs, feelings, and behaviors related to classroom language learning arising from the uniqueness of the language learning process.” Foreign language anxiety is associated with three related anxieties, involving the following: communication apprehension, test anxiety, and fear of negative evaluation (Horwitz et al., 1986). MacIntyre and Gardner (1994a, p. 284) have defined anxiety as the “feeling of tension and apprehension specifically associated with second language contexts, including speaking, listening, and learning.” According to the latter, language anxiety is a form of anxiety that is essentially stimulated by situational factors (speaking in front of the class, tests, or even being called on by the teacher. Young (1991) claims that language anxiety is instigated by six interconnected factors that are interrelated in terms of three distinct aspects: the learner, the teacher, and the instructional practice. The six sources of language anxiety involve (a) personal and interpersonal anxiety (b) learner beliefs regarding language learning (c) instructor beliefs on language learning (d) instructor-learner interactions (e) classroom procedures, and (f) language testing. Fundamentally, all previously noted causes of foreign language anxiety act as a determent to language acquisition.

Fundamentally, even though researchers of the field have described the nature and causes of anxiety in various ways, the literature unanimously maintains that anxiety, along with negative emotion may become evident and develop into an impediment during language learning (Cheng, 2004; Atay and Kurt, 2006; Kurt and Atay, 2007). In particular, empirical research has validated that anxiety hinders performance (i) academically, as it is associated with low academic achievement (ii) cognitively, as anxiety may act as an affective filter impeding information from entering the cognitive processing system, thus prone to influence both speed and learning accuracy and (iii) personally, as language learning may become an unpleasant or even traumatic experience (Zheng, 2008).

### Foreign language (FL) writing anxiety

Writing anxiety, as a term, refers to writers who are competent enough to intellectually adhere to a task, but, nonetheless, face difficulty with the process of writing production (Zheng, 2008). In particular, foreign language writing anxiety has been classified as a specific type of anxiety, closely related to the language-particular skill of writing (Matsuda, 2003), and a negative, anxious feeling that fundamentally, disrupts the writing process (McLeod, 1987). Language writers facing writing anxiety are led toward feelings of anguish and detestation toward the writing process (Madigan et al., 1996); have difficulty in the production of effective and logical pieces of writing (Aitman, 1985), difficulties that range from the writing of straightforward letters to multifaceted reports. Procrastination, apprehension, tension, low self-esteem, lack of motivation, withdrawal, and avoidance have all been cited as problems related to the production of an assigned writing task (Cheng, 2004). Studies have shown that students with high levels of writing anxiety wrote shorter compositions and qualified their writing less than their low-anxiety counterparts did (Hassan, 2001). What's more, studies have shown that English Second Language (ESL) writing anxiety can have profound effects on both ESL writing performance (Cheng, 2004) and writing quality (Veit, 1980). However, studies have also shown that there are ways in which Foreign language writing anxiety can be tackled, as instructional practice has shown to be connected to students' foreign language classroom anxiety levels. Hence, various studies have centered on investigating ways in which instructional practices could combat the writing anxiety levels of ESL writers.

Schweiker-Marra and Marra (2000) focused on the effects of prewriting activities on the writing performance and anxiety levels of at risk fifth-grade students. The subjects were supported through a writing program that utilized pre-writing activities so as to investigate whether anxiety levels would lessen and written expression would improve. The results of the study noted improvement for the experimental group as anxiety levels lessened and written expression improved. The experimental group, more so than the control group felt less anxiety in regards to writing and believed that less effort was required from them during the writing process as a result of the instruction provided and emphasis placed on prewriting activities. A similar study conducted by Worde (2003) attempted to identify the factors that contribute to anxiety as well as the factors that may reduce anxiety as identified by students themselves. Interview questions focused on the participants' beliefs, experiences as well as feelings. The Foreign Language Classroom Anxiety Scale (FLCAS; Horwitz et al., 1986) was utilized so as to obtain a comprehensive picture of the sources of anxiety in a language classroom. The results of the study elicited that 73% of the subjects were anxious students of which 34% were rated as highly anxious. The study confirmed previous research that has shown that anxiety impedes foreign language production and achievement; it also confirmed that reducing anxiety may enhance learners' motivation (Young, 2008). Essentially, however, the study under discussion provided valuable evidence as it elaborately depicted students' perceptions as to the factors that may reduce their feelings of anxiety. That is, students stressed that textbook changes as well as teachers' pedagogical practices and idiosyncrasies are factors that could lead to reduced language learning anxiety. Specifically, the subjects stressed the necessity of a methodology that would outline and highlight scaffolded learning and in turn reinforce the material so as to aid comprehension and retention. Ozturk and Cecen (2007) have investigated the effects of portfolios on the writing anxiety levels of students. The latter based their hypothesis on empirical research highlighting the significance of portfolio keeping in foreign language teaching. The Second Language Writing Anxiety Inventory (SLWAI; Cheng, 2004) a background questionnaire and two reflective sessions were the means of data collection. The findings of the study confirmed that portfolio keeping is beneficial in assisting L2 learners to overcome writing anxiety as well its benefits as a teaching practice that would willingly be adopted by all perspective (100%) language teachers. Fundamentally, the research to date is not only concerned in investigating why language learners face such high levels of foreign language writing anxiety, but it is also concerned as to how they can tackle it. However, one important aspect that has not been investigated to date involves the effects of metacognitive or self-regulation writing skill development on foreign language learners' anxiety levels.

### Anxiety and academic performance

Anxiety is significant within the field of cognition and performance as it is often associated with adverse effects on the performance of cognitive tasks. Specifically, anxiety has been shown to have negative effects on working memory capacity, imposing a direct threat to performance (Eysenck et al., 2007). Working memory has been defined as “those mechanisms or processes that are involved in the control, regulation and active maintenance of task-relevant information in the service of complex cognition” (Miyake and Shah, 1999, p. 450). Basically, working memory is the small amount of information that can be held in the mind and used in the execution of cognitive tasks. In antithesis, long-term memory is the vast amount of information saved in one's life (Cowan, 2014). Working memory coordinates information received via the senses and retrieved from long-term memory, for tracking, storage, and manipulation (Logie, 1999).

Working memory is an extensively used term in psychology as it influences learning in several ways. It has been repeatedly associated to intelligence, information processing, executive function, comprehension, problem solving, and learning. Hence, researchers of the field have highlighted the importance of training working memory so as to enhance learning and education. Specifically, it has been advocated that by adapting the materials with the working memory abilities of the learner, learning, and education are both enhanced. Specifically, some of the aspects of working memory that are likely to develop involve: capacity, speed, knowledge, and the use of effective strategies (Cowan, 2014, p. 197).

Furthermore, even though there have been various theoretical explanations discussing the adverse effects of anxiety on performance, this paper will concentrate on the processing efficiency theory by Eysenck and Calvo (1992) that has evolved over time. Processing efficiency theory makes two vital distinctions that include both task effectiveness and task efficiency. Task effectiveness essentially deals with the quality of task performance and is described through standard behavioral measures such as response precision. Efficiency, in turn, involves the association between performance effectiveness and the individuals' endeavors involved in task performance. Specifically, processing efficiency declines due to usage of additional resources to achieve the desired performance level. Based on the processing efficiency theory, anxiety is an aversive emotional and motivational state occurring in threatening circumstances. Specifically, state anxiety is interactively determined by trait anxiety or test anxiety and situational stress (Eysenck and Calvo, 1992; Derakshan and Eysenck, 2009); therefore, individuals in an anxious state frequently worry and feel intimidated about a current goal. In many cases, they attempt to develop effective strategies to reduce anxiety so as to achieve the goal. However, processing efficiency theory argues that, essentially, anxiety has negative effects on cognitive tasks because it places significant demands on cognitive resources. Based on the processing efficiency theory, worry is a component of state anxiety and directly responsible for the effects of anxiety on performance effectiveness and efficiency. Worry is activated in stressful circumstances and usually involves individuals with high trait anxiety. Worry has two effects. Firstly, it involves cognitive interference, that is, worrying thoughts consume the limited attentional resources of working memory, and attentional resources thus become less available for simultaneous task processing. Secondly, worry involves increased motivation to reduce the anxiety state. Individuals who are able to engage in augmented effort and make usage of supplementary processing resources such as strategies, are able to reduce their anxiety state. However, when auxiliary resources are inaccessible, performance is threatened (Eysenck et al., 2007). Ultimately, as previously mentioned when the working memory abilities of the learner are successfully adapted to the material, then the learning is enhanced.

The second assumption of processing efficiency theory involves the mechanisms and components of working memory that are affected by anxiety. Specifically, processing efficiency theory assumes that the main effects of worry and anxiety in general are on the central executive. The central executive, based on Baddeley's (1986) working memory system, plays the most fundamental role of the working memory system. It involves the processing of information and having self-regulatory functions such as performance monitoring, planning, decision-making, and strategy selection. The negative effects of anxiety on performance and efficiency should be greater with tasks that cause significant demands on the processing and storage capacity of working memory. That is, individuals' worrisome thoughts interfere with processing and storage function. This way, there is an added burden on the self-regulatory mechanism obstructing such thoughts and producing auxiliary processing activities. Essentially, there is evidence supporting that anxiety (state or trait) is closely associated with performance impairments on a plethora of tasks. A meta-analysis based on hundreds of studies revealed an overall correlation on −0.29 between test anxiety and academic achievement (Hembree, 1988). However, there is also evidence to support that the heavy demands that anxiety places on cognitive resources could be tackled through metacognitive writing skill development.

### Metacognitive writing skill development

Metacognitive knowledge has been widely researched during the last two decades as it has been found to play a vital role in human learning and performance as well as writing skill development. Empirical studies have emphasized the importance of teaching metacognitive strategies to improve students' writing (Harris and Graham, 1999; Perry and Drummond, 2002; Harris et al., 2003) as studies have shown that metacognitive processes play a vital role in writing proficiency development (Garcia-Sanchez and Fidalgo-Rodendo, 2006). The most common conceptualization that has emerged defines metacognition as the knowledge and control individuals have over their own cognition and learning experiences (Flavell, 1979). Metacognition has been described as an individual's capacity to reflect, monitor and regulate ones' thinking processes (Ruan, 2005, p. 106), and is of vital importance as students are able to become independent and autonomous learners (Englert et al., 1988). Ghonsooly and Ghanizadeh (2011) posit that theories and practices that deal with metacognitive knowledge have been widely applied to the learning process, and that, basically, it is through metacognitive knowledge that students are able to mobilize, direct and sustain their instructional efforts.

The role of metacognitive knowledge in writing is acknowledged in most current models of composing either explicitly or implicitly (Flower and Hayes, 1981; Bereiter and Scardamalia, 1987; Hayes, 1996). Although there are differences between various theoretical definitions, students with metacognitive regulation are generally characterized as active, efficiently managing their own learning through monitoring and strategy use (Winne and Perry, 2000; Zimmerman, 2000). Students with a metacognitive regulation plan are able to set goals, organize, self-monitor, and self-evaluate at various points during the acquisition process. This way, learners are self-aware, knowledgeable and decisive regarding the way in which they approach writing. Pintrich (2000) has described metacognitive regulation as a constructive process wherein learners set goals on the basis of both their past experiences and their present environments. Fundamentally, metacognitive regulated learning mediates the associations between learner characteristics, context, and performance (Pintrich, 2000).

Empirical studies have emphasized the importance of metacognitive skills to improve students' writing (Harris et al., 2003), as studies have shown that metacognitive processes play a vital role in writing proficiency development (Garcia-Sanchez and Fidalgo-Rodendo, 2006). Donovan and Bransford (2005) have posited that a metacognitive approach aids students in developing the ability to take control of their own learning, to determinedly define learning goals, and to monitor their progress in achieving them. Metacognition also deals with self-generated thoughts, feelings, and actions that are used to achieve personal goals (Zimmerman, 2000). Students that have metacognitive knowledge engage in goal-setting, vigilantly choose suitable strategies to accomplish a task, generate self-instructions to complete a task successfully, utilize time management, choose effective environmental settings, monitor progress, and performance evaluation, and also request assistance from appropriate sources when required (Zimmerman, 1998).

The provision of metacognitive skill development enables learners to become self-regulated writers: not only able to accomplish a given task but also having gained an enhanced faith in their capabilities as writers. Most importantly, however, learners are supported in taking control of and self-regulating their own learning processes. Collins et al. (1989) argue that metacognitive processes are the organizing principles of expertise, particularly in domains such as writing. In a similar manner, Bereiter and Scardamalia (1987) argue that the compositional conduct of writers who do not possess metacognitive strategies can be resembled to that of novice or young writers approaching language in a knowledge-telling fashion. That is, being unaware that expert writing involves organizing one's ideas about a topic, elaborating goals to be achieved in the writing, thinking about the audience and so on. Expert writers, on the other hand, approach writing in a knowledge-transforming fashion, that is, they plan what they are going to write and revise what they have written (Bereiter and Scardamalia, 1987). In essence, the aforementioned authors assumed that children might have appropriate self-regulatory mechanisms available but they fail to use them. One of the reasons is due to working memory limitations.

### Procedural facilitation

Cognitive psychology has vigilantly investigated the role of working memory as it is indispensable in writing, and has described working memory as the limitations that individuals experience when executing tasks entailing the use of memory. That is, working memory's limit in regards to the amount of information it can withhold, as well as the length of time it can withhold this information. When limited working memory resources are utilized for the execution of various writing processes, they make the processes interfere with one another. Bereiter and Scardamalia (1987), proposed the provision of procedural facilitation as a way to decrease the executive burden of writing, thereby increasing writing expertise in knowledge tellers so that they could fundamentally gradually become knowledge transformers.

Bereiter and Scardamalia (1987) proposed the implementation of special supportive procedures that would provide cues or routines for switching into and out of new regulatory mechanisms, while keeping the executive procedures, as a whole, intact, and minimizing the resource demands of the newly added self-regulatory mechanisms. Procedural facilitation engages modeling, cognitive, metacognitive and self-regulatory processes, and consists of four steps: (i) identify a self-regulation function that appears to work in expert performance (ii) describe the self-regulatory function in terms of mental operation as explicitly as possible (iii) create cues or routines that minimize demands of mental resources (iv) provide external supports or teachable routines for reducing the information-processing burden of mental operations. Fundamentally, procedural facilitation, deals with the provision of special supportive procedures, to assist novice writers in becoming expert writers; essentially it assists in combating working memory limitations.

### The cognitive apprenticeship approach in writing

The cognitive apprenticeship approach in writing has been widely investigated as it has shown fruitful outcomes (Zimmerman and Kitsantas, 2002; Rijlaarsdam et al., 2005; Graham and Perin, 2007; Kellogg, 2008). The principles of cognitive apprenticeship are characterized by learning through observing rather than learning by doing (Kellogg, 2008) and highlight the principles of social learning through mentor observation (Roggoff, 2008). Cognitive apprenticeship lies on the features of Vygotsky's (1978) concept of the zone of proximal development, in which the learner's focus is placed on tasks that extend their capacities so as to obtain a further level of development. Essentially, the provision of cognitive apprenticeship approach in writing, fosters training programs, in which a mentor offers assistance to the learner, so that they are enabled to successfully deal with the demands of the task at hand (Roggoff, 2008). The principles of cognitive apprenticeship are based on the development of interventions that both train and instruct writers, while also providing writing instructors the method to successfully train writers to effectively use their knowledge during the composing process. Cognitive apprenticeship training programs aim to train writers to retrieve and use what they know during composition, as dictated by the use of prior knowledge principle; for example, directing learners' attention on a sub-goal such as preparing an outline.

Through cognitive apprenticeship, the learners' attention is focused on the mentor's behavior rather than being involved in cognitive processes and motor execution (Rijlaarsdam et al., 2005). Learning to write an effective piece of writing is a difficult task, one that requires an extensive range of knowledge that must be available in long-term memory. This knowledge is accessed either by rapidly retrieving it from long-term memory or by actively maintaining it in short term memory. At the same time, writers are called upon to make a range of decisions such as “what to say,” content, and “how to say it,” rhetoric. These decisions, along with mechanics, impose high demands on both the writer's attention, the cognitive system of thinking, and other resources of working memory. Moreover, heavy demands are placed on working memory by planning, sentence generation, and reviewing processes. However, cognitive apprenticeship training programs, with a focus on deliberate practice, might best assist writers gain sufficient executive attention to provide a high degree of cognitive control so that they are able to carry on the various representations of the text such as planning, writing, and reviewing. Essentially, cognitive apprenticeship programs train and instruct writers how to gain executive control over cognitive processes so that they can respond to the needs of the task at hand (Kellogg, 2008). Collins et al. (1989) argue that methods of cognitive apprenticeship (cognitive and metacognitive expertise) be adapted to the teaching and learning of cognitive skills. This way, interplay between observation, scaffolding, and increasingly independent practice will aid learners to the following: develop self-monitoring and correction skills and integrate the skills and conceptual knowledge needed to advance toward expertise. Various studies that have implemented cognitive strategy instruction writing models to date have shown that metacognitive knowledge is directly interrelated to enhanced performance.

### Cognitive strategy instruction (SRSD)

A significant advancement in educational psychology is cognitive strategy instruction (Pressley et al., 1990). Cognitive strategies have been defined as the “cognitive processes that the learner intentionally performs to influence learning and cognition. Examples include basic processes, such as using a rehearsal strategy to memorize a list, and metacognitive strategies, such as recognizing whether one comprehends a passage” (Mayer, 2001, p. 86). Cognitive strategies aim to “design and validate” strategies that would ultimately serve to enhance the learning and performance of students with learning difficulties (Wong et al., 2003) as well as all other students, whether gifted, average, handicapped, experiencing difficulties in schoolwork, receiving special educational services, or even those who will find them useful to achieve advanced school performance (Pressley, 2002).

The Self-Regulated Strategy Development (SRSD; Graham and Harris, 1989) writing instructional model is one of the most researched writing programs to date (Tracy et al., 2009), demonstrating writing improvements for students of various ages and abilities (Schnee, 2010). The SRSD was implemented in a foreign language setting for the purpose of this study. The SRSD (Graham and Harris, 2005) assists learners in developing fundamental writing production strategies (planning, organizing, revising, self-regulation procedures) and fosters the formation of positive attitudes about students' writing and their writing abilities (Harris, 1982).

The Harris and Graham's SRSD model (Graham and Harris, 1996, 2005) is a multi-component writing instruction model that has validated its effectiveness through empirical evidence and has a dynamic empirical basis for its effectiveness. The SRSD model of instruction has shown improvements in writing that have been determined as maintained over time and generalized across setting, genres, people, and media (e.g., paper and pencil to word processor; Graham and Harris, 2005; Santangelo et al., 2008). The SRSD promotes the following key-elements of instruction on: (i) higher order cognitive and metacognitive processes taught through explicit strategies instruction (ii) enhancement of students' positive writing attitudes and elimination of negative personal behavior (iii) explicit self-regulatory procedures such as goal-setting, self-monitoring, self-evaluation, and self-reinforcement (iv) a flexible and adaptable writing strategy meeting teachers' needs in a mainstream classroom setting, with new strategies introduced as well as the upgrading of previously taught strategies (Graham and Harris, 2003) (v) assists the writing needs of students with and without learning difficulties (vi) a criterion-based instruction providing students the time they require to produce superior writing outcomes (vii) interactive learning between teacher and students from the dialectical-constructivist viewpoint (Pressley et al., 1992) (viii) individualized instruction tailored to students' needs and capabilities (Graham and Harris, 2003). Essentially, the SRSD assists educators in fostering positive environments that stimulate students' active learning while reinforcing academic achievement that, in turn, fosters positive behavior and endorses learning.

What's more, a meta-analysis by Graham and Perin (2007) concerning writing intervention literature that involves students in grades four (4) through twelve (12), with an emphasis on experimental and quasi experimental studies, demonstrated that explicit and systematic strategy instruction highly impacted students' overall writing quality. That is, the SRSD showed to have a strong and positive effect on the quality of students' writing with an average weighted effect size of 1.14. The SRSD, to date, has the largest weighted effect size of any other writing intervention examined.

### Rational, purpose, and hypotheses of the study

The existing literature to date argues that foreign language writing anxiety is in essence, a universal phenomenon that ultimately hinders foreign language writing achievement. Hence, both educators and researchers share concerns over the negative effects of writing anxiety on language learning. The reason and importance of this study is to offer guidance for intervention programs designed to decrease writing anxiety in second/foreign language acquisition. For this reason, the purpose of this study was to implement a writing intervention program so as to reduce the writing anxiety levels of foreign language learners. The initial hypothesis, thus, is that by applying an effectively designed learning environment, foreign language anxiety would lessen. Specifically, it was hypothesized that (i) in the initial measurement all three subscales of anxiety measured, somatic, cognitive, and behavioral, would characterize both the control and the experimental group (ii) the control group that followed the traditional program, would show no change in foreign language writing anxiety with regards to the factors of somatic anxiety, cognitive anxiety and behavioral anxiety (iii) the experimental group would show lower foreign language writing anxiety levels with regards to all three factors of anxiety, a result that can largely be attributed to the content of the intervention program applied (iv) that the experimental group would show lower anxiety levels than the control group in post-test, a result that could be largely attributed to the content of the intervention program applied (v) the specific intervention program applied would indicate acceptable validity and reliability indices for the Greek population.

## Methods

### Procedure

Following approval by the Ethics Committee, access to students in public schools was requested. Standard consent procedures were followed prior to questionnaire administration. Students were informed about the purpose of the study, the assessment, and the procedure of data collection. They were reassured that their responses would be kept strictly confidential, and that they would only be used for research purposes. The students were asked to voluntarily participate, completed a consent form prior to questionnaire completion, and were tested according to classroom group, as further on explained.

### District demographics

The participants of this study were enrolled in two public primary schools located in the region of Chania, on the island of Crete, in Greece. The two public schools were randomly selected, of which one served as the control group, and one as the experimental group. The school that served as the control group consisted of one-hundred (100) grade five (5), and grade six (6) students, and was a suburban school located West of the city of Chania. The school that served as a research group consisted of seventy-seven (77) grade five (5), and six (6) students, and was also a suburban school North of the city of Chania.

### Participants

A sample of one hundred and seventy-seven (177) students (*n*_boys_ = 88 and *n*_girls_ = 89), volunteered to participate in the study. Respondents were randomly drawn from two mainstream primary schools in the city of Chania, Crete, and were all of Greek background. Students' age ranged from eleven (11) to twelve (12) years (*M*_age_ = 11.53 years, *SD* = 0.56). Ninety (90) students (50.8%), were enrolled in grade five (5), and eighty-seven (87) students (49.2%), were enrolled in grade six (6). The students were randomly assigned to a control, and an experimental group.

### Instructional setting

A total of eight (8) groups participated for the purpose of this study. One school consisted of two (2) grade-five (5), and two (2) grade six (6) groups, and served as the experimental group. The other school consisted of two (2) grade-five (5), and two (2) grade-six (6) groups, and served as the control group. The classrooms consisted of twenty (20), to twenty-five (25) students.

Grade five (5), and grade six (6) students were selected on the basis that their writing abilities could be investigated in greater detail, and a clearer impression of their writing profiles could be obtained. That is, students of these grade levels would be able to discuss their learning strategies, and complete the anxiety questionnaire provided for the purpose of this study. None of the subjects had previously participated in a writing course. Specifically, Greek EFL primary school students attend 3 h of English lessons per week, in which all four language skill areas are practiced: writing, listening, reading, and speaking. The duration of each lesson is 45 min. A course book designed by the Pedagogical Institute guides the course of each lesson.

The instructional approaches of both the experimental, and the control group were similar in nature in terms of teaching writing. That is, even though both educators verified the importance of writing as a skill to be acquired, it was determined that explicit writing instruction was not a component of the teaching methodology, and that students were infrequently asked to engage in writing tasks. In particular, neither the control group, nor the experimental group were taught planning, or revising strategies. Writing skills such as: handwriting, spelling, punctuation, grammar-drills, vocabulary, and syntax through fill in the blank exercises characterized the traditional writing instruction procedure for both, the control, and the experimental group.

### Instrumentation

The SLWAI (Cheng, 2004) was used in the present study. The SLWAI has been depicted as a language skill-specific anxiety scale as it has shown higher correlation with writing achievement (Cheng et al., 1999).

The SLWAI consists of 22 items, and is designed to assess the level of anxiety that the students feel regarding second language writing. The SLWAI's 22 items constitute the following three qualities that typify the SWAI's factors: (a) somatic anxiety, (b) avoidance behavior, and (c) cognitive anxiety. Somatic anxiety, refers to one's perception of the physiological effects of the anxiety experience as reflected in increased “autonomic arousal, and unpleasant feeling states, such as nervousness, and tension. Avoidance behavior, refers to displaying an avoidance pattern toward writing, and cognitive anxiety deals with negative expectation, fear or worry of negative evaluation, and tests” (Morris et al., 1981, p. 541). Each of the SLWAI subscales consist of seven items, and students' responses to the items were provided based on a 5-point Likert type format with anchors ranging from *strongly agree* (1) to *strongly disagree* (5). Also, a global score was included in the analysis so as to better comprehend the concept of anxiety from a holistic perspective (Cheng, 2004). The higher the score obtained by the subscales, and the total score of the SLWAI are indicative of a higher level of writing anxiety.

### Intervention

The SRSD writing instructional model was integrated into the regular classroom. The researcher undertook the complete instruction of all eight (8), English language classes of both the control, and the experimental group so as to integrate the SRSD model of instruction. In particular, two grade five (5) classes, and two grade six (6) classes were part of the experimental group. Similarly, two grade five (5) classes, and two grade six (6) classes were part of the control group. Seventeen weeks of instruction took place for the completion of the study. The initial week served for the investigator and students to get acquainted, and for the administration of pre-tests. Fifteen weeks of instruction followed, and 1 week of post-test administration. In particular, 45 min sessions, of 45 min of instruction took place.

Participants were divided into two treatment groups; an experimental group, and a control group. During pre-test both the control and the experimental groups writing skills were examined in regards to two writing genres. The two writing genres examined included story writing, and expository writing. Assessment for story writing, involved writing a paper in response to a writing prompt. However, as a means to increase motivation, students were also given the option of writing a story of their own choice. The writing prompt that involved story writing, depicted line drawings of children, and animals engaged in an activity. The subjects were asked to use their imagination to write a story. In regards to expository writing, writing prompts included questions asking students their opinion on school or home issues (e.g., Should children be allowed to choose their own pets? Should students help in the household chores?). In regards to post-test, the control group received no instruction whatsoever, and followed the traditional English program as outlined by the ministry of Education. The experimental group however, received explicit structured strategy based procedural facilitation guided by the SRSD writing instructional model (Harris and Graham, 1986; Graham and Harris, 1989) that is discussed as follows.

### SRSD instruction

The experimental group was provided with procedural facilitation through the implementation of the SRSD model (Graham and Harris, 1996, 2005), on two genre specific writing strategies “WWW, What = 2, How = 2,” for short story writing, and “TREE” for expository essay. The experimental group was also provided with both the general writing strategy, in which these two strategies were embedded POW, and the indispensable accompanying knowledge, and self-regulatory procedures required to use these strategies. Through procedural facilitation, navigated by the SRSD model, the experimental group was offered explicit, and systematic strategy instruction so as to accomplish specific writing tasks. The experimental group, was aided with additional information or skills (vocabulary, grammar-drill instruction, good word choice, interesting openings etc.), required for the utilization of these strategies (Graham and Harris, 1996, 2005). Procedural facilitation, navigated by the SRSD writing approach, aspired to explicitly teach self-regulation procedures (goal-setting, self-monitoring, self-instructions, self-reinforcement), and to assist students in administering the target strategies, and writing task. Fundamentally, procedural facilitation, navigated by the SRSD writing approach, aspired to assist students attain solid, and evident, or visible confirmation of their writing progress.

The implementation of the framework consists of six flexible, or adoptable stages (develop background knowledge, discuss it, model it, memorize it, support it, independent performance), which provides broad-spectrum guidelines that can be re-ordered, combined or modified to foster learners', and educators' needs (Graham and Harris, 2005). The self-regulation framework involves six elements: goal setting, self-assessment, self-instruction, self-reinforcement, imagery, and managing the writing environment (Santangelo et al., 2008).

The effects of two genre specific writing strategies “WWW, What = 2, How = 2,” and “TREE” as well as the general writing strategy embedded “POW” were examined. Correspondingly, the accompanying knowledge and self-regulatory procedures, required to apply the above mentioned strategies were also examined. Procedure for strategy based procedural facilitation included: mnemonic charts, flashcards, graphic organizers, transition words, million dollar words, transfer sheets, self-statements, the use of technology, and role-play. Integrated language skills were taught in conjunction, through the use of writing activities, and included: reading, speaking, and listening. That is, students enhanced all three language skills (reading, speaking, listening, grammar, and vocabulary), in conjunction with each other through the provision of explicit writing instruction, and procedural facilitation on writing production. The communicative approach was fostered so as to engage participants in meaningful tasks, and enhance motivation (McDonough and Shaw, 1993). Explicit, intensive, and scaffolded instruction on generating ideas, and include basic genre-specific elements to produce the two writing genres (story, expository essay) were applied. Instruction centered on the following stages that included: choosing a topic, considering purpose, identifying audience, gathering, and organizing ideas.

### Data analysis

Statistical analysis was divided in two phases. In the first phase, in order to examine the *a priori* factor validity of the SLWAI for Greek students, confirmatory factor analytic methods were applied. Items' univariate distributional properties, such as univariate skewness, univariate kurtosis were examined for possible retention, whereas the Mardia's coefficient of multivariate normality was evaluated in order to select the factor analysis method (Bollen, 1989; West et al., 1995). Based on the SLWAI factor structure (Cheng, 2004), instrument's items were uniquely allowed to load only the hypothesized factors (Bentler, 1995). The following fit indices were used for estimating the sufficiency of the measurement models: (1) Chi-square (χ^2^) (Bentler and Bonett, 1980), (2) Satorra-Bentler χ^2^/*df* ratio (Bentler and Bonett, 1980; Byrne, 1994; Bentler, 1995), (3) *Non-Normed Fit Index* (NNFI; Bentler and Bonett, 1980; Byrne, 1994), (4) *Comparative Fit Index* (CFI; Byrne, 1994; Marsh, 1994; Bentler, 1995), (5) *Standardized Root Mean Squared Residual* (SRMR; Hu and Bentler, 1995; Tabachnick and Fidell, 2006), and (6) *Root Mean Squared Error of Approximation* (RMSEA) and the 90% CI of the RMSEA (Steiger, 1990; Hu and Bentler, 1999). Values of NNFI, CFI, and RCFI above 0.900 represent an acceptable fit of the model (Bentler, 1990), whereas, when SRMR and RMSEA values are close to or lower than 0.050 demonstrate an acceptable fit (Steiger, 1990; Tabachnick and Fidell, 2006). Hu and Bentler (1999) proposed the cut-off criterion of SRMR for acceptable fit close or below to 0.080 and for RMSEA 0.060. Based on the instrument's factor structure, items' factor loadings should exceed the cut-off criterion of 0.400.

The internal consistency for each SLWAI subscale was estimated based on the: (a) means and range (min–max) of item values, inter-item and item-total correlations, and (b) Cronbach's *a* coefficient. For an acceptable internal consistency, Cronbach's *a* coefficient should exceed 0.70 (Tabachnick and Fidell, 2006).

A 2 (Conditions: experimental, control) × 2 (Time: PRE, POST) multivariate repeated analysis of variance (RMANOVA) was conducted to examined the main purpose of the study. In the following, based on the results of the RMANOVA, separate univariate analysis were performed on each of the three SLWAI factors (somatic anxiety, avoidance behavior, cognitive anxiety) examining students' differences between-subjects conditions (experimental *vs*. control), as well as, the within-subject repeated (pre–post) measures. Based on the results of Mauchly's test of sphericity, if the assumptions of sphericity were not met in the within—subjects' variable analyses, the *F* estimation was based on the Green-House Geisser correction and the respective degrees of freedom (Tabachnick and Fidell, 2006; Field, 2009). *T*-tests followed any significant between and within effects testing pairwise comparisons, applying Bonferroni adjustment. The significance level was set at *p* < 0.05. The Statistical Package for Social Sciences (SPSS 21.0 Win) was used for the statistical analyses.

## Results

### Factor structure of the second language writing anxiety inventory (SLWAI)

#### Confirmatory factor analysis

The examined distributional properties of the SLWAI items aimed to determine the level of data normality. SWAI items indicated low values of skewness and kurtosis (skewness_range_ = −1.338 to 0.587, kurtosis_range_ = −1.309 to 2.735; West et al., 1995). In addition, the Mardia's (1970) coefficient of multivariate normality (normalized estimate = 9.613) revealed acceptable multivariate kurtosis (lower than 528, based on the formula *p* (*p*+2) where *p* equals the number of observed variables; Mardia, 1970; Bollen, 1989). Based on the above, the maximum likelihood method was chosen in the subsequent confirmatory factor analysis, as a method that is appropriate in normally distributed data.

The results of the hypothesized three-factor correlated model (FM_3C_) of the SLWAI indicated an adequate fit to the data (Table 1). The χ^2^ was 298.41 (*p* < 0.001, *df* = 204), the Satorra-Bentler χ^2^ was 274.05, and the χ^2^*/df* ratio value supported an acceptable fit to the data (χ^2^ */df* ratio = 1.46), as it was lower than two (Byrne, 1989). The NNFI was 0.91, the CFI 0.92, and the RCFI 0.93 exceeding the cut-off criterion of 0.90 of acceptable fit. Also, the SRMR reached the cut-off criterion of 0.080, whereas the RMSEA successfully attained the criterion of 0.06 (RMSEA = 0.05, 90% CI of the RMSEA = 0.04 to 0.06; Hu and Bentler, 1999). The items loadings values ranged from 0.39 to 0.74 (mean factor loadings = 0.60). The average off-diagonal standardized residual was 0.06, supporting that the examined model fits the data. The SLWAI latent factor correlations were for somatic anxiety—avoidance behavior 0.68 (*p* < 0.001), for somatic anxiety—cognitive anxiety 0.86 (*p* < 0.001), and for avoidance behavior—cognitive anxiety 0.54 (*p* < 0.001).

**Table 1:** **Three factors parameters estimate for the SLWAI items**.

**Items**	**Skewness**	**Kurtosis**	**Item loadings**	**Item uniqueness**
1	−0.09	−0.61	0.43	0.90
2	−0.32	2.74	0.39	0.92
3	−0.34	1.89	0.50	0.87
4	−0.45	−1.22	0.42	0.91
5	0.59	−0.89	0.69	0.73
6	−0.48	−0.78	0.51	0.86
7	−0.42	−0.50	0.48	0.88
8	−0.48	−0.02	0.50	0.86
9	−1.22	1.72	0.73	0.68
10	−0.50	−0.95	0.76	0.65
11	−0.89	0.93	0.63	0.77
12	−0.14	−1.31	0.69	0.72
13	−0.86	1.08	0.73	0.68
14	−0.48	−0.56	0.71	0.71
15	−0.72	−0.11	0.73	0.68
16	0.43	−0.73	0.74	0.68
17	−1.06	0.92	0.63	0.78
18	−0.97	0.56	0.42	0.91
19	−0.25	−0.40	0.54	0.85
20	−0.62	−0.15	0.66	0.75
21	−1.26	0.95	0.65	0.76
22	−0.65	0.91	0.55	0.84

Two alternative measurement models were tested for further examination of SLWAI factor structure. For this purpose, initially a single factor model was set, specifying that all items loaded on a single factor (FM_1_), and secondly, a three uncorrelated factors model (FM_3U_) was examined. The SLWAI single factor measurement model (FM_1_) revealed a poor fit to the data (Table 2), since the NNFI and CFI did not reach the cut-off criterion of 0.90. Additionally, the RMSEA, as well as χ^2^*/df* ratio (χ^2^*/df* ratio = 2.42) values were high. In the same vein, the SLWAI three uncorrelated factors model (FM_3U_) didn't fulfill the criteria of acceptable fit. The NNFI, CFI, and RCFI indices were lower than 0.90, criterion of acceptable fit, whereas the SRMR was high. The fit indices of the three tested measurement models are presented in (Table 2).

**Table 2:** **Fit indexes of the three-factor and alternate SLWAI CFA models**.

	**χ^2^**	***d**f*	**Δχ^2^**	**Δ*_df_***	**NNFI**	**CFI**	**SRMR**	**RMSEA**	**90% CI RMSEA**	**AIC**	**CAIC**
FM_3C_	298.41	204	–	–	0.91	0.92	0.07	0.05	0.04–0.06	−109.59	−961.53
FM_3U_	544.94	209	246.54	5	0.70	0.73	0.22	0.100	0.09–0.11	126.94	−745.87
FM_1_	506.41	209	208.00	5	0.74	0.76	0.08	0.090	0.08–0.10	88.41	−784.41

*χ^2^, chi-square statistic; df, degrees of freedom; Δχ^2^, χ^2^ difference; Δ_df_, df difference; CFA, Confirmatory Factor Analysis; NNFI, Non-Normed Fit Index; CFI, Comparative Fit Index; SRMR, Standardized Root Mean Residual; RMSEA, Root Mean Squared Error of Approximation; CI, Confidence Interval; AIC, Akaike's Information Criterion; CAIC, Consistent Akaike's Information Criterion*.

#### Internal consistency

The Cronbach's *a* indices of the SLWAI factors indicated that all coefficients were internally consistent (somatic anxiety = 0.77, avoidance behavior = 0.80, cognitive anxiety = 0.82). Further support to the reliability of the SWAI subscales was provided through the inter-factor correlations, as well as, the item-factor correlations (Table 3).

**Table 3:** **Internal consistency indices (mean, minimum value, maximum value) for the SLWAI subscales**.

**SLWAI subscales**	**Inter-item correlations**	**Item-total correlations**	***a***
	**Mean (Min-Max)**	**Mean (Min-Max)**	**Cronbach**
Somatic anxiety	0.33 (0.18–0.57)	0.49 (0.35–0.64)	0.77
Avoidance behavior	0.36 (0.16–0.59)	0.54 (0.33–0.66)	0.80
Cognitive anxiety	0.36 (0.21–0.55)	0.54 (0.39–0.63)	0.82

### Preliminary analysis

#### Data screening

Univariate and multivariate distribution properties were examined, prior to main analyses (Tabachnick and Fidell, 2006). Skewness and kurtosis values of the examined SLWAI items were low (skewness _range_ = −0.77 to 0.21, kurtosis _range_ = −1.55 to 0.47). Additionally, the assumption of equality of covariance matrices was satisfactory at the univariate level (Levene's test), however it was violated at the multivariate one (Box's *M*-test). Therefore, as the appropriate multivariate test statistic the Pillai's Trace was chosen due to its robustness over test violations (Tabachnick and Fidell, 2006; Field, 2009).

### Main analysis

#### Differences between experimental and control conditions

Initially, the students' baseline (pre-test) SLWAI responses (somatic anxiety, avoidance behavior, cognitive anxiety) between the experimental and control group were examined using multivariate analysis of variance (MANOVA). No significant differences revealed on the SLWAI subscales between the students participating in the experimental and control groups, Pillai's Trace *V* = 0.023, *F*_(1, 175)_ = 1.34, *ns*, $\eta_{\text{p}}^{2}$ = 0.02.

A repeated measure multivariate analysis of variance (RMANOVA) was performed to examine whether the participants SLWAI responses changed across time (pre–post) in the experimental conditions. A significant Condition (2) × Time (2) interaction was revealed, Pillai's Trace *V* = 0.826, *F*_(1, 175)_ = 272.89, *p* < 0.001, $\eta_{\text{p}}^{2}$ = 0.83, on SLWAI subscales. Also, a significant Condition, Pillai's Trace *V* = 0.501, *F*_(1, 175)_ = 57.93, *p* < 0.001, $\eta_{\text{p}}^{2}$ = 0.50, and Time, Pillai's Trace *V* = 0.811, *F*_(1, 175)_ = 247.37, *p* <.001, $\eta_{\text{p}}^{2}$ = 0.81, main effects were appeared. Descriptive statistics (means, standard deviations) of the SLWAI subscales for the experimental and control conditions across the two time measures are presented in (Table 4).

**Table 4:** **Means (***M***), Standard Deviations (***SD***) of the SLWAI subscales**.

**SLWAI subscales**	**Experimental group**		**Control group**	
	**Pre ***M***** **(*SD*)**	**Post ***M***** **(*SD*)**	**Pre ***M***** **(*SD*)**	**Post ***M***** **(*SD*)**
Somatic anxiety	3.91 (0.64)	2.01 (0.45)	3.74 (0.58)	3.77 (0.62)
Avoidance behavior	3.46 (0.84)	2.42 (0.38)	3.24 (0.76)	3.40 (0.64)
Cognitive anxiety	4.00 (0.71)	1.88 (0.32)	3.85 (0.59)	3.92 (0.54)

Subsequent analysis supported a significant Condition × Time interaction for somatic anxiety, *F*_(1, 175)_ = 554.28, *p* < 0.001, $\eta_{\text{p}}^{2}$ = 0.76. Examining somatic anxiety variation (pre–post) in each experimental condition, a significant decrease revealed in the experimental group, *t*_(76)_ = 27.29, *p* < 0.001. However, no significant changes over time appeared for the control group, *t*_(99)_ = −0.68, *ns*. In the pre-test measure, no significant differences revealed between students in the control and the experimental conditions, *t*_(175)_ = −1.80, *ns*, while in the post-measure students participating in the experimental condition revealed significantly lower somatic anxiety compared to those in the control group, *t*_(175)_ = 22.05, *p* < 0.001.

With regard to the avoidance behavior subscale, the results supported a significant Condition × Time interaction, *F*_(1, 175)_ = 129.04, *p* < 0.001, $\eta_{\text{p}}^{2}$ = 0.42. In more detail, from pre to post measure a significant decrease was detected in the experimental condition, *t*_(76)_ = 11.37, *p* < 0.001, while in the control condition avoidance behavior slightly increased, *t*_(99)_ = −2.65, *p* < 0.01. In addition, no significant differences were revealed in the initial measure between students in the two conditions, *t*_(175)_ = −1.76, *ns*, while in the post-measure the experimental condition students revealed significant lower avoidance behavior compared to those in the control group, *t*_(175)_ = 12.73, *p* < 0.001.

A significant Condition × Time interaction was detected for the cognitive anxiety, *F* = 658.25, *p* < 0.001, $\eta_{\text{p}}^{2}$ = 0.79. In the experimental condition, a significant change over time was appeared, *t*_(76)_ = 26.67, *p* < 0.001, indicating a decrease of cognitive anxiety. However, in the control group no significant changes were shown between the pre and post measures, *t*_(99)_ = −1.55, *ns*. Finally, in the pre-test, no significant differences were revealed between control and experimental conditions, *t*_(175)_ = −1.46, *ns*, while in the post measure the students participating in the experimental condition revealed significant lower cognitive anxiety compared to those in the control group, *t*_(175)_ = 31.49, *p* < 0.001.

## Discussion

Research has supported the existence of foreign language writing anxiety as an additional affective variable that debilitates the learning and/or the effectiveness of foreign language writing (Yan and Horwitz, 2008; Horwitz et al., 2010). Empirical research has also validated that writing anxiety has been shown to negatively affect writing quality, and that students faced with writing anxiety have difficulty in the production of effective and logical pieces of writing (Veit, 1980; Aitman, 1985). Foreign language writing is a demanding task that requires the application and continuous interaction of numerous language abilities, in addition to general metacognitive abilities. Foreign language writing also requires the integration and application of multiple sub-skills, which operate at different processing levels (Coker, 2007). The developing nature of linguistic and metacognitive knowledge and fluency, or the accessibility to this linguistic knowledge, is what fundamentally makes foreign language writing such a strenuous task (Schoonen et al., 2003; Ruan, 2005). These demands create an “extra burden that overwhelms the limited capacity of short-term memory” (Flower and Hayes, 1981, p. 373), and thus rationalize the differences found between expert and inexpert writers' writing processes and writing products. In addition, emotional strain may be activated during anxiety driven situations and may act as an additional impediment for the cognitive processes that occur during the learning process, leading students to isolation owing to difficulties as regards to decision-making and withdrawal from resolving these difficulties (Vassilaki and Vamvoukas, 1997). However, through the provision of environments fostering metacognitive skill development, the cognitive demands of writing could be minimized, and writing expertise could be developed; consequently, anxiety levels could successfully be tackled.

The findings of this study supported the initial hypothesis that important statistical differences would be found between the experimental group and the control group following the provision of metacognitive skill development through explicit strategy based instruction that fosters procedural facilitation and cognitive apprenticeship. Particularly, as was expected, the experimental groups anxiety levels lessened following the implementation of the intervention program applied. The results of the study, therefore support the findings that, following metacognitive skill development in a strategy based-procedural facilitative environment, the participant's anxiety levels decreased.

More specifically, the initial measurement results of the SLWAI (Cheng, 2004) as regards to all three subscales of anxiety measured-somatic anxiety, cognitive anxiety, and behavioral anxiety-were found to describe both groups of participants (control and experimental). However, cognitive anxiety, which deals with negative expectation, fear or worry of negative evaluation and test, was found to be the most common type of ESL writing anxiety experienced by young Greek foreign language writers. These findings corroborate Cheng's (2004) conclusion that cognitive anxiety is closely related to test anxiety or negative evaluation, and could have a great influence on L2 writing production. Students with test anxiety, or fear of negative evaluation, experience cognitive anxiety interference, and have difficulties focusing on the writing task at hand. As regards to learners' behavioral anxiety, in the initial measurement, both groups of participants also reported experiencing avoidance behavior for writing English or avoiding situations that require writing in English. Avoidance behavior, a negative consequence of ESL writing anxiety, thus, would result in hindering L2 writing improvement. ESL writing anxiety was also shown to have negative effects and trigger physical symptoms such as accelerated heartbeat, perspiration, or even blushing that are the negative effects of behavioral anxiety, and further impede the writing progress.

These findings corroborate with previous findings stating that cognitive anxiety could negatively influence writing production (Cheng, 2004). That is, the findings of this study confirm previous conclusions stating that the cognitive and the affective domain complement one another (Dewaele, 2005; Marcos-Llinas and Garau, 2009) and corroborate previous empirical research that has shown that anxiety may hinder writing production, cognitively, by acting as an affective filter that impedes information from entering the cognitive processing system (Zheng, 2008).

Moreover, the findings of this study indicated a negative impact of anxiety on language learning achievement, corroborating previous findings (MacIntyre and Gardner, 1989, 1991a,b). In particular, following the writing intervention program applied, the experimental groups anxiety levels, especially as regards to cognitive anxiety lessened. The findings, thus, sustain previous conclusions stating that anxiety has negative effects on working memory capacity, and imposes a direct threat on writing tasks (Eysenck et al., 2007). Moreover, the findings of this study support previous studies that have shown that the effects of foreign language writing anxiety prevents learners from their academic achievements (Aida, 1994; Zheng, 2008) as they are associated with feelings of apprehension, tension, low self-esteem, lack of motivation, procrastination (Petzel and Wenzel, 1993), anguish, and detestation for the writing process (Madigan et al., 1996).

The findings of this study, support the debilitating effects of anxiety on language learning (Horwitz, 2000); specifically, the findings support Horwitz's (2001) depiction of foreign language anxiety as a negative emotional reaction to language learning. The findings also confirm previous research conducted that has determined anxiety to be an affective variable that inhibits the learning and/or the production of a second or foreign language (Horwitz et al., 2010).

The findings also corroborate with previous studies that have highly investigated the positive outcomes of cognitive strategy instruction (Wong et al., 2003) and cognitive apprenticeship training programs that focus on deliberate practice and have shown to assist writers in acquiring writing skills (Graham and Perin, 2007; Kellogg, 2008) as well as reducing executive control problems (Bereiter and Scardamalia, 1987). The findings also support studies that have shown that procedural facilitation reduces executive control problems (Bereiter and Scardamalia, 1987) and assists novice ESL learners' in decreasing writing demands (Cumming and So, 1996). Fundamentally, the findings support studies that have shown that cognitive apprenticeship training programs that focus on deliberate practice basically assist writers' in acquiring thinking and problem-solving writing skills (Kellogg, 2008).

Metacognitive strategies have shown to have positive effects on students' L1 writing. However, limited empirical research to date has focused attention on the provision of training frameworks that emphasize metacognitive strategy instruction (Horwitz et al., 1986), so as to minimize young foreign language learners cognitive load and, consequently, lower anxiety levels. The plethora of studies to date have focused attention on adult ESL/EFL university undergraduate or postgraduate students (Roca de Larios et al., 2002). Few studies have investigated the second/foreign language writing processes of young school children and how they relate to anxiety levels. The present study has come to fill this gap in the literature. The importance of this study lies on the fact that the findings validate strategy based-procedural facilitative writing environments as an effective instructional practice as the results show that foreign language writing anxiety is reduced when language skill development is enhanced. Specifically, the importance of this study is (a) to lessen EFL learners' writing anxiety levels (b) through the development of metacognitive skills for young EFL students (c) to stress the writing production process as a meaning making activity (d) to provide an environment that gradually offers learning control to young EFL learners and (e) to suggest a coherent way to offer explicit strategy-based instruction through a procedural facilitative environment; one that offers proper scaffolding that leads to learner autonomy, and metacognitive skill development (Tsiriotakis, 2013). At a more practical level, the findings of this study hope to assist and inspire ESL educators to reach informed decisions concerning the necessity of intervention programs that are designed to decrease writing anxiety levels in second/foreign language acquisition.

This study, essentially, highlights the importance of transforming students into active entities who are in control of their own learning processes, who embrace dynamic problem solving skills, who develop true expertise in order to make their journey throughout the learning process both visible and meaningful (Collins et al., 1989), ultimately, overcoming their writing anxiety levels. This paper also hopes to increase awareness that academic failure could be due to working memory limitations (Cowan, 2014). Empirical research has validated that writing involves multiple representations and processes, with limitations in working memory shown to constrain skill development. However, following metacognitive skill development, executive attention can effectively coordinate multiple writing processes and representations.

The results of this study hope to provide inference for both theory and pedagogy in terms of the significance of metacognitive strategy instruction in EFL writing for all foreign language writers. Essentially, a new approach highlighting metacognitive strategy instruction guided by procedural facilitation that promotes cognitive apprenticeship is proposed for implementation in foreign language settings so that writers are more equipped to tackle the demands of foreign language writing with lower anxiety levels.

Young English foreign language learners take up a large percentage of the population and the English language, the officially lingua franca (Graddol, 2006), is a vital instrument of school success and most importantly future success. Hence, it is vital to foster the instructional needs of all young foreign language learners and equip them with the necessary strategies to deal with the intricacies of foreign language learning. This study could be replicated in another country in which English is also being taught as a foreign language so as to determine whether the findings are peculiar to Greece. Finally, as foreign language reading is a prerequisite for the development of foreign language writing (Cumming, 2006), an investigation of the effects of metacognitive strategy training programs, on the reading performance and reading anxiety levels of young learners is recommended for future research.

## Limitations of the study

This study has certain limitations. Initially, the examination of a follow-up measurement could not be conducted as all sixth (6th) grade subjects were graduating from grade school on that same year. The following year, all subjects enrolled in various public high schools across the region of Chania, on the island of Crete, in Greece. For this reason, it was not possible to find all subjects and conduct a follow up study which would have provided valuable information as to the effects of maintenance of acquired strategies on the subjects' anxiety levels. Another notable limitation of the study was that all subjects included in the study were of Greek ethnicity. As such, caution must be used in generalizing the results. Further research on strategy instruction and anxiety effects across a range of cultures is required so as to corroborate the effects and to substantiate the effectiveness of the SRSD writing instructional model on the writing anxiety levels of English foreign language learners. Finally, the questionnaire utilized to measure the students' writing anxiety levels might give useful information about the impact of foreign language writing anxiety. However, it seems not to provide enough evidence of the students' actual writing performance, indicating the need for further research. Despite the limitations of this study, the findings are promising and highlight the necessity of future research studies to assist students with the demands of foreign language learning.

## Author contributions

The tasks of IT included: the research hypothesis, data entry, analysis, and interpretation of data, theory formulation, literature review and discussion section. The tasks of EV included: the theory formulation, literature review and discussion section. The tasks of IS included the theory formulation, literature review and discussion section. The tasks of NS included: the research hypothesis, data entry, analysis, and interpretation of data.

### Conflict of interest statement

The authors declare that the research was conducted in the absence of any commercial or financial relationships that could be construed as a potential conflict of interest.
